# Oryeongsan inhibits LPS-induced production of inflammatory mediators via blockade of the NF-kappaB, MAPK pathways and leads to HO-1 induction in macrophage cells

**DOI:** 10.1186/1472-6882-14-242

**Published:** 2014-07-14

**Authors:** You-Chang Oh, Yun Hee Jeong, Jeong-Ho Ha, Won-Kyung Cho, Jin Yeul Ma

**Affiliations:** 1 Korean Medicine (KM)-Based Herbal Drug Development Group, Korea Institute of Oriental Medicine, Daejeon, Yuseong, Republic of Korea

**Keywords:** Oryeongsan, Inducible nitric oxide synthase, Heme oxygenase-1, Nuclear factor-kappaB, Mitogen-activated protein kinase

## Abstract

**Background:**

Oryeongsan (OR) is an herbal medication used in east-Asian traditional medicine to treat dysuresia, such as urinary frequency, hematuria, and dysuria due to renal disease and chronic nephritis. Recent studies showed that protective effect against acute gastric mucosal injury and an inhibitory effect on the renin-angiotensin-aldosterone pathway of OR. However, its effect on inflammation still remains unknown. In this study, to provide insight into the biological effects of OR, we investigated their effects on lipopolysaccharide (LPS)-mediated inflammation in the RAW 264.7 macrophage cells.

**Methods:**

We investigated the pharmacological and biological effects of OR on the production of pro-inflammatory cytokines, inflammatory mediators, and related products through Enzyme-linked immunosorbent assay (ELISA), reverse transcription-polymerase chain reaction (RT-PCR) and Western blot analysis. Also, we examined the activation and suppression of nuclear factor (NF)-kappaB and mitogen-activated protein kinases (MAPKs) pathways in LPS-stimulated macrophages via Western blot analysis in order to explore inhibitory mechanism of OR.

**Results:**

OR had anti-inflammatory effects by inhibiting the production of nitric oxide (NO), tumor necrosis factor (TNF)-alpha, interleukin (IL)-6, and IL-1beta. In addition, it strongly suppressed cyclooxygenase (COX)-2 and inducible nitric oxide synthase (iNOS), NO synthesizing enzymes. It also induced heme oxygenase (HO)-1 expression and inhibited NF-kappaB signaling pathway activation and phosphorylation of MAPKs.

**Conclusions:**

We further demonstrate the anti-inflammatory effects and inhibitory mechanism of OR in LPS-stimulated macrophages for the first time. OR contains strong anti-inflammatory activity and affects various mechanism pathways including NF-kappaB, MAPKs and HO-1. Our results suggest that OR has potential value to be developed as an inflammatory therapeutic agent from a natural substance.

## Background

OR is a traditional ancient herbal medication in East Asia. It is a prescription described in the Sanghanron, an ancient Chinese medical book. OR is composed of five medicinal herbs including Alisma Rhizome, Atractylodes Rhizome White, Chuling, Poria and Cinnamon Bark. OR is currently prescribed for the treatment of edema, dizziness, vomiting and symptoms associated with renal disease. A previous study demonstrated that OR protects against ethanol-induced acute gastric mucosal injury [1]. Another study revealed that OR has an inhibitory effect on the renin-angiotensin-aldosterone system in rats [2]. Additionally, recent studies have shown that amelioration of streptozotocin diabetes-induced renal damage by OR [3]. It has also been reported that OR exert protective effect on adriamycin-induced nephrotic syndrome in rats [4]. However, the effect of OR on inflammation remains unknown.

Inflammation is a normal physiological immune response to protect body from infection or tissue injury and results in activation of various immune cells such as macrophages, neutrophils, and lymphocytes. In normal state, inflammatory mediators, such as NO and inflammatory cytokines, generated from macrophage cells take an essential role in host survival and tissue repair [5]. However, these inflammatory mediators are overexpressed by certain stimuli and could cause autoimmune and inflammatory diseases [6-8].

Macrophages play an important role in the regulation of inflammation and immune responses [6,9]. Specific stimuli such as LPS which is endotoxin from gram-negative bacteria, give rise to activation of macrophages. Activated macrophages secrete inflammatory mediators such as NO and prostaglandin (PG)E_2_ and produce inflammatory cytokines such as TNF-α and IL-6 [5,10]. These inflammatory mediators and cytokines are essential for host survival after infection, and are necessary for the recovery of tissue damage [5]. NO and PGE_2_ are synthesized by iNOS and COX-2, respectively, and iNOS expression is closely related to the induction of HO-1.

HO-1 is a stress-inducible protein that catalyzes the oxidative degradation of heme. Two other isoforms exist: HO-2 and HO-3 [11]. HO-1 expression is enhanced not only by free heme, but also by various pro-inflammatory stimulants such as NO, LPS, cytokines, heavy metals, and other oxidants [12,13]. Biliverdin is rapidly transformed into bilirubin, which decreases NO production and iNOS expression in murine macrophages stimulated with LPS [14,15]. Carbon monoxide, another product of heme degradation by HO, inhibits NO secretion and reduces inflammation. Thus, enhanced HO-1 production may result in the reduction of iNOS expression and decrease the amount of free radicals [16].

Among the cytokines, TNF-α, IL-6 and IL-1β are important factors involved in the progression of many inflammatory diseases. These cytokines can be regulated by activation of the transcription factor NF-κB. NF-κB is composed of homo or heterodimeric combinations of NF-κB/Rel proteins, including Rel (cRel), RelA (p65), RelB, NF-κB1 (p50) and NF-κB2 (p52) [17]. The main inducible form is consisting of the p65 and p50 subunit. NF-κB plays an important role in the expression of inflammatory genes, and is involved in the pathogenesis of rheumatism and other chronic inflammatory diseases [18]. In unstimulated state, NF-κB is present in the cytoplasm attached to the suppressor protein inhibitor of NF-κB alpha (IκBα), but specific stimulants such as LPS give rise to free NF-κB through degradation and phosphorylation of IκBα [19]. Activated NF-κB is translocated from the cytoplasm to the nucleus, then binds to the promoter and induces the expression of various inflammatory genes including iNOS, COX-2, inflammatory cytokines, and chemokines [20,21]. Previous studies showed that nuclear translocation of NF-κB promotes the transcription of iNOS, COX-2 and inflammatory cytokines including TNF-α and IL-6 [22]. Most anti-inflammatory agents reduce the expression of inflammatory factors via inhibition of NF-κB activity [23].

The MAPK signaling pathway plays an important role in relaying inflammatory information from the extracellular space to the cytoplasm and nucleus [24]. There are at least three known pathways of MAPK, such as extracellular signal-regulated kinase (ERK), p38 and c-Jun NH_2_-terminal kinase (JNK) MAPK. Activated ERK can phosphorylate various transcription factors; p38 and JNK constitute a part of the stress response pathway activated by various stimulants induced by specific factors [25]. MAPK is activated by phosphorylation, and subsequently induces the activation of NF-κB pathway and expression of iNOS gene. A previous study demonstrated that specific MAPK inhibitors can reduce iNOS gene expression [26]. In the present study, we evaluated the inhibitory effect of OR on inflammation induced by LPS in RAW 264.7 macrophages. Furthermore, we investigated whether OR-induced modulation of NF-κB and MAPK signaling pathways and their influence on HO-1 induction were responsible for the anti-inflammatory effects of OR.

## Methods

### Materials and reagents

Roswell Park Memorial Institute (RPMI) 1640 medium, fetal bovine serum (FBS), and antibiotics were purchased from Lonza (Basel, Switzerland). LPS and bovine serum albumin (BSA) were obtained from Sigma (St. Louis, MO, USA). A cell-counting kit (CCK) was purchased from Dojindo Molecular Technologies, Inc. (Kumamoto, Japan). Various primary and secondary antibodies for Western blot analysis were purchased from Cell Signaling Technology, Inc. (Boston, MA, USA). ELISA antibody sets for cytokine detection were obtained from eBioscience (San Diego, CA, USA). RNA extraction and DNA synthesizing kits were purchased from iNtRON (Sungnam, Korea) and Bioneer (Daejeon, Korea), respectively. Oligonucleotide primers were synthesized by Bioneer (Daejeon, Korea). The standard compounds cinnamic acid and cinnamaldehyde were purchased from Sigma (St. Louis, MO, USA), and atractylenolid III was purchased from Chem Faces (Wuhan, China). Marker compound purity, as determined by high-performance liquid chromatography (HPLC), was higher than 98%. HPLC grade acetonitrile and trifluoroacetic acid (TFA) were purchased from J. T. Baker Inc. (Philipsburg, NJ, USA). Distilled water (DW) was filtered through a 0.45-μm membrane filter from ADVANTEC (Tokyo, Japan) before analysis.

### Preparation of herbal decoction OR

OR is composed of five medicinal herbs listed in Table 1. All herbs were purchased from Yeongcheon Herbal Market (Yeongcheon, Korea). All voucher specimens were deposited in an herbal tank, placed in 15,000 mL of DW and then extracted by heating for 3 h at 115°C and under high pressure (Gyeongseo Extractor Cosmos-600, Inchon, Korea). After extraction, the solution was filtered using standard testing sieves (150 μm) (Retsch, Haan, Germany), freeze-dried and kept in desiccators at 4°C before use. The acquisition was 347 g and the yield was 23.2%. The freeze-dried extract powder was then dissolved in DW, centrifuged at 14000 rpm for 10 min and supernatant was filtered (pore size, 0.2 μm) and kept at 4°C prior to use.

**Table 1:** Herbal components and amount of OR decoction

**Herbs**	**Amount of herbs (g)**
Alisma Rhizome	500
Atractylodes Rhizome White	300
Chuling	300
Poria	300
Cinnamon Bark	100

### Cell culture and drug treatment

RAW 264.7 cells were obtained from the Korea Cell Line Bank (Seoul, Korea) and grown in complete RPMI 1640 medium. The cells were incubated in a humidified 5% CO_2_ atmosphere at 37°C. To stimulate the cells, the medium was exchanged with fresh RPMI 1640 medium, and LPS (200 ng/mL) was added in the presence or absence of OR (10, 100, 500, or 1000 μg/mL) for the indicated periods.

### Cell viability assay

Cytotoxicity was analyzed using a CCK. OR was added to the cells and incubated for 24 h at 37°C with 5% CO_2_. CCK solutions were added to each well and the cells were incubated for another 1 h. Then the optical density was read at 450 nm using an ELISA reader (Infinite M200, Tecan, Männedorf, Switzerland).

### Measurement of NO production

NO production was analyzed by measuring nitrite in the supernatants of macrophages incubated with or without OR. The cells were pretreated with OR and stimulated with LPS for 24 h. Griess reagent (1% sulfanilamide, 0.1% naphthylethylenediamine dihydrochloride, and 2.5% phosphoric acid) was added to the cultured supernatant and incubated at room temperature (RT) for 5 min [27]. The absorbance was read at 570 nm.

### Determination of cytokine production

The secretion of inflammatory cytokines TNF-α, IL-6 and IL-1β was analyzed using an eBioscience mouse ELISA antibody set (San Diego, CA, USA). The inhibitory effect of OR was determined by an ELISA reader at 450 nm absorbance.

### Western blot analysis

Expression of various proteins was evaluated by Western blot analysis according to standard procedures. The cells were pretreated with OR and stimulated with LPS for the indicated periods at 37°C. After incubation, the cells were harvested and resuspended in radio immunoprecipitation assay (RIPA) lysis buffer (Millipore, Bedford, MA, USA) with protease and phosphatase inhibitor cocktail (Roche, Basel, Switzerland). After cell debris was discarded following centrifugation, protein concentration was determined using Bradford’s reagent and equal amounts of protein were subjected to sodium dodecyl sulfate-polyacrylamide gel electrophoresis (SDS-PAGE). After transferring the proteins onto a nitrocellulose membrane (Millipore, Bedford, MA, USA), the membrane was blocked with 3% BSA in Tris-buffered saline with 0.1% Tween 20 (TBS-T). Then the membrane was incubated with each primary antibody at 4°C overnight and subsequently incubated with HRP-conjugated secondary antibodies. Specific proteins were detected using SuperSignal West Femto Chemiluminescent Substrate (Thermo Scientific, Rockford, IL, USA).

### Preparation of cytosolic and nuclear extracts for NF-κB detection

Cytosolic and nuclear fractions were isolated using NE-PER Nuclear and Cytoplasmic Extraction Reagents (Thermo Scientific, Rockford, IL, USA) according to the procedure described by the manufacturer. The fractions were stored at −80°C before use.

### RNA extraction and RT-PCR

Total RNA was isolated using an easy-BLUE™ RNA extraction kit (iNtRON, Daejeon, Korea) according to the procedure described by the manufacturer. The total RNA was transformed into cDNA using AccuPower® CycleScript RT PreMix (Bioneer, Daejeon, Korea). Specific primers amplified by PCR are described in Table 2. The following PCR conditions were applied for TNF-α, IL-6, IL-1β, COX-2, iNOS, HO-1, and β-actin: 35 cycles of denaturation at 94°C for 30 s, annealing at the temperature indicated in Table 2 for 30 seconds, and extension at 72°C for 30 seconds [27-31].

**Table 2:** Primers used for RT-PCR analysis

**Target gene**	**Primer sequence**	**Annealing temp**
TNF-α	F: 5′-AGCACAGAAAGCATGATCCG-3′	55°C
	R: 5′-GTTTGCTACGACGTGGGCTA-3′	
IL-6	F: 5′-CATGTTCTCTGGGAAATCGTGG-3′	58°C
	R: 5′-AACGCACTAGGTTTGCCGAGTA-3′	
IL-1β	F: 5′-TGCAGAGTTCCCCAACTGGTACATC-3′	64°C
	R: 5′-GTGCTGCCTAATGTCCCCTTGAATC-3′	
COX-2	F: 5′-CACTCAGTTTGTTGAGTCATTC-3′	45°C
	R: 5′-GATTAGTACTGTAGGGTTAATG-3′	
iNOS	F: 5′-AGCCCAACAATACAAATGACCCTA-3′	56°C
	R: 5′-TTCCTGTTGTTTCTATTTCCTTTGT-3′	
HO-1	F: 5′-TGAAGGAGGCCACCAAGGAGG-3′	62°C
	R: 5′-AGAGGTCACCCAGGTAGCGGG-3′	
β-actin	F: 5′-ATGAAGATCCTGACCGAGCGT-3′	58°C
	R: 5′-AACGCAGCTCAGTAACAGTCCG-3′	

F, forward; R, reverse.

### Preparation of standard solutions and samples

Standard stock solutions of atractylenolid III, cinnamic acid and cinnamaldehyde were prepared by dissolving 0.2 mg each standard in 1 mL 60% methanol to yield a final concentration of 200 μg/mL. To prepare analytical samples, 10 mg OR extract in 1 mL DW was extracted by ultra-sonication and filtered through a 0.2-μm syringe membrane filter from Whatman Ltd. (Maidstone, UK) before injection into the HPLC system for analysis. All standard stock and sample solutions were stored at −4°C in a refrigerator before analysis.

### Chromatographic conditions

The HPLC-DAD system (Hitachi, Tokyo, Japan) consisted of a pump (L-2130), autosampler (L-2200), column oven (L-2300) and UV/VIS diode array detector (L-2455). The output signal of the detector was recorded using EZChrom Elite software for Hitachi. For sample analysis, an OptimaPak C_18_ column (4.6 × 250 mm, 5 μm; RS Tech Co., Daejeon, Korea) was used, and the column oven temperature was kept at 35°C. The injection volume was 20 μL, and the flow rate of the mobile phase was 1.0 mL/min. The wavelength of the UV detector was set at 220 nm. The mobile phase was water containing 0.1% TFA and acetonitrile, with gradient elution at a flow rate of 1.0 mL/min (Table 3).

**Table 3:** HPLC conditions used for the analysis of OR

**Item**	**Condition**		
**Mobile phase**	**Time (min)**	**Water (containing 0.1% TFA)**	**Acetonitrile**
	0	90	10
	5	90	10
	40	10	90
	40	0	100
	60	0	100
Flow rate	1.0 mL/min		
Inject volume	20 μL		
Column	OptimaPak C_18_ (4.6 × 250 mm, 5 μm, RS tech Co., Daejeon, Korea)		
Column temperature	40°C		
UV wavelength	220 nm		

The English in this document has been checked by at least two professional editors, both native speakers of English. For a certificate, please see:

http://www.textcheck.com/certificate/7WNmoy.

### Statistical analysis

The results are expressed as mean ± standard error (SE) for all experiments. Statistical significance was determined by Student’s t-tests after comparing each treated group to the negative control. Each experiment was repeated at least three times to yield comparable results. Values of P < 0.01 and P < 0.001 were considered statistically significant.

## Results

### Effects of OR on cell viability and NO production

**Figure 1:**
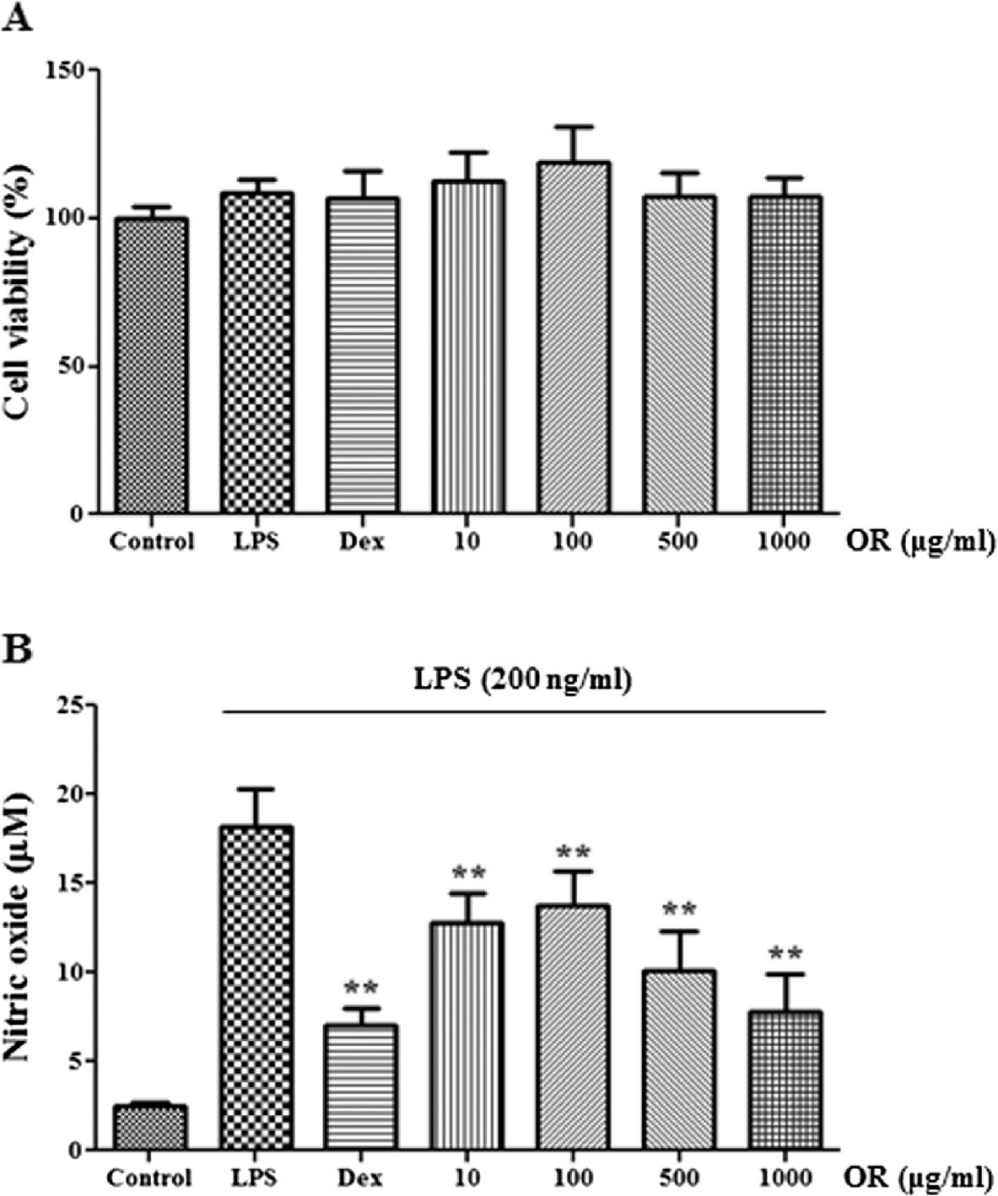
**Effect of OR on (A) cell viability and (B) NO production induced by LPS stimulation.** RAW 264.7 cells were pretreated with OR for 30 min before 24 h incubation with LPS. **(A)** The cytotoxicity was determined using CCK. **(B)** The culture supernatant was analyzed for nitrite production. As a control, the cells were incubated with vehicle alone. Data represent mean ± SE values of duplicate determinations from three independent experiments. *p < 0.01 and **p < 0.001 were calculated from comparisons with the LPS-stimulation value.

We first examined OR cytotoxicity at concentrations of 10–1000 μg/mL in macrophages. As shown in Figure 1A, OR was not cytotoxic up to 1000 μg/mL, indicating that OR is not toxic to macrophages. Because NO inhibition can relieve inflammation, we examined the inhibitory effect of OR on NO generation in RAW 264.7 cells upon LPS stimulation. Dexamethasone, a well-known anti-inflammatory drug, was used as a positive control. As presented in Figure 1B, OR concentration-dependently suppressed NO secretion with statistical significance. In particular, OR (1000 μg/mL) inhibited NO secretion to a similar extent as observed with the positive control.

### Inhibitory effect of OR on LPS-induced TNF-α, IL-6 and IL-1β production

**Figure 2:**
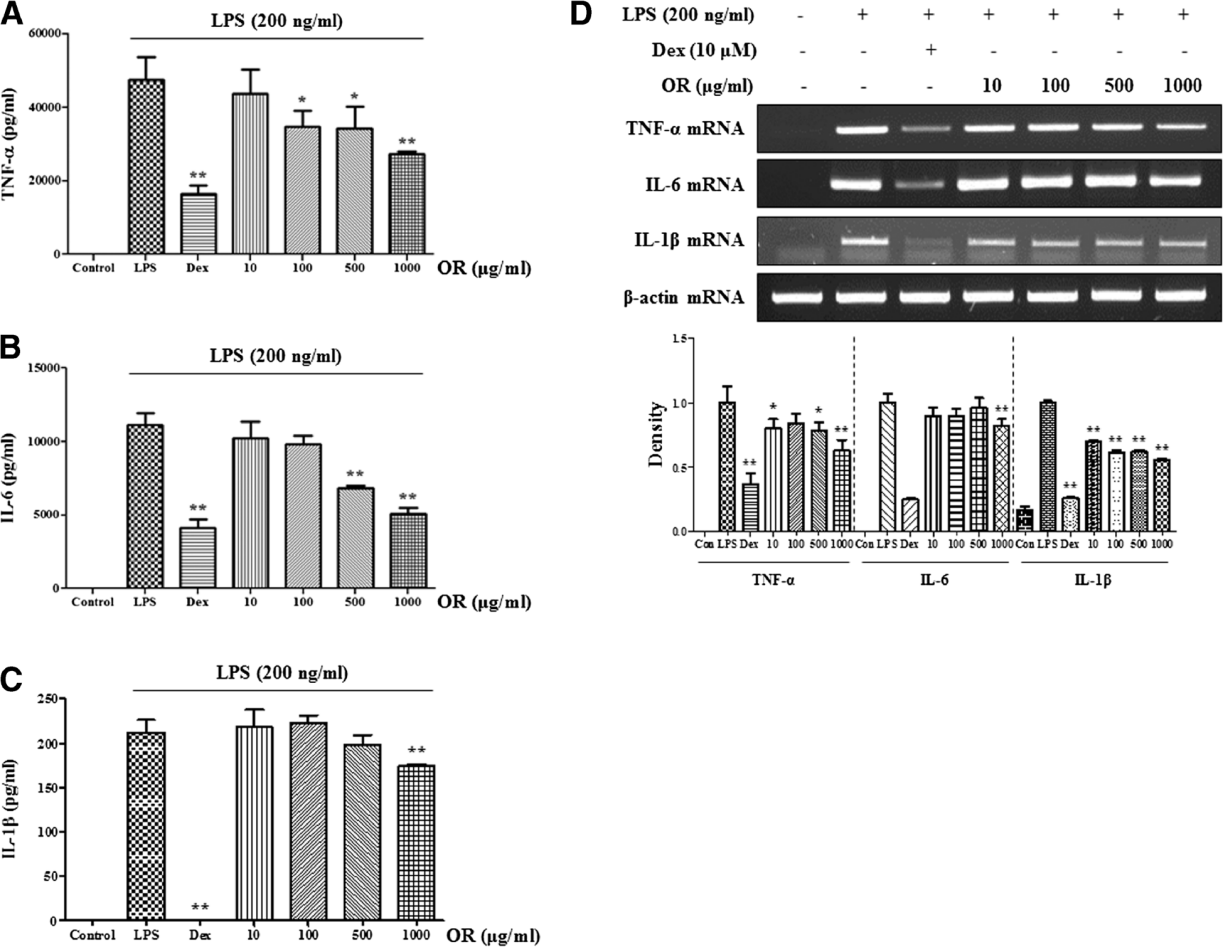
**Effect of OR on the expression of (A–C) cytokines and (D) mRNAs.** Cells were pretreated with OR for 30 min before being incubated with LPS for **(A–C)** 24 h and **(D)** 6 h. Cytokine production was measured by ELISA and mRNA level was analyzed by RT-PCR. RNA value was quantitated using an i-MAX^TM^ Gel Image Analysis System (Core Bio, Seoul, Korea). Data represent the mean ± SE values of duplicate determinations from three independent experiments. *p < 0.01 and **p < 0.001 were calculated by comparison with the LPS-stimulation value.

We examined the inhibitory effect of OR on the expression of TNF-α, IL-6 and IL-1β cytokines, other inflammatory mediators. Cytokine expression was analyzed using ELISA and RT-PCR analysis. TNF-α cytokine and mRNA were inhibited by OR treatment in a concentration-dependent fashion (Figure 2A and D). Consistent with the TNF-α result, OR also inhibited IL-6 cytokine production concentration-dependently (Figure 2B). However, the inhibitory effect of OR on IL-6 mRNA expression was negligible, with the exception of the effect of the 1000 μg/mL concentration (Figure 2D). By contrast, OR slightly inhibited IL-1β cytokine production and strongly repressed IL-1β mRNA expression (Figure 2C and D).

### Inhibitory effect of OR on LPS-induced COX-2 and iNOS expression, and OR effect on HO-1 induction

**Figure 3:**
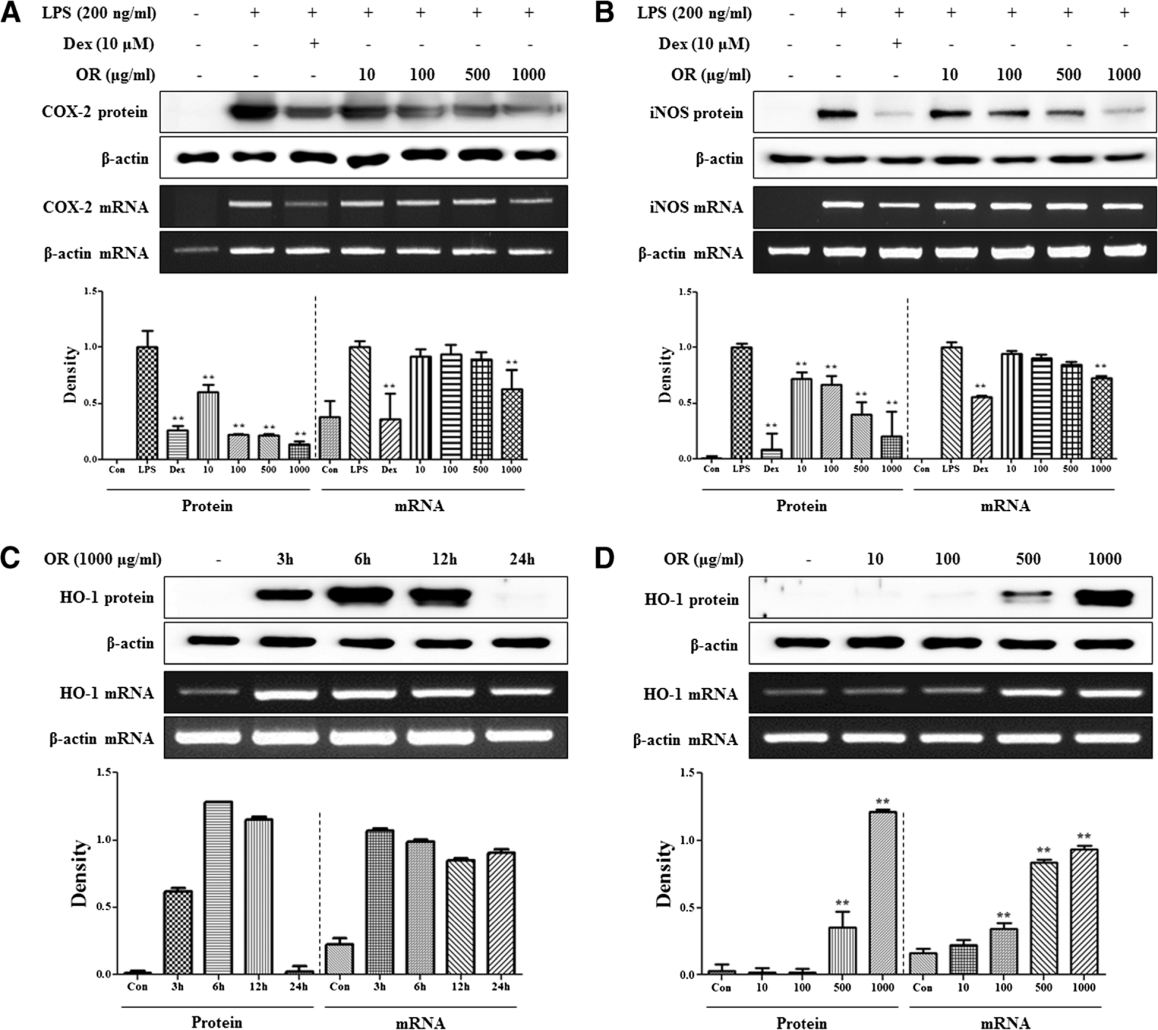
**The effects of OR on (A) COX-2, (B) iNOS, (C, D) HO-1 in macrophages.** The cells were treated with **(A, B)** LPS alone or with LPS and OR for 24 h and **(C, D)** OR alone for the indicated periods. Protein levels were determined by Western blot analysis, as described in the Materials and Methods, and were quantitated using a Davinch-chemi^™^ Chemiluminescence Imaging System CAS-400SM (Core Bio, Seoul, Korea). mRNA expression was analyzed by RT-PCR. Data represent mean ± SE values of duplicate determinations from three independent experiments. *p < 0.01 and **p < 0.001 were calculated from comparisons with the LPS-stimulation value.

COX-2 and iNOS are the synthesizing enzymes of PGE_2_ and NO, respectively. Therefore, COX-2 and iNOS expression were next investigated using Western blot and RT-PCR. As presented in Figure 3A and B, OR suppressed both protein and mRNA expression of COX-2 and iNOS. In particular, expression of COX-2 and iNOS protein was significantly inhibited in a concentration-dependent manner. We also examined HO-1 induction in OR-treated macrophages, which is known to contribute to the inflammatory response. Western blot and RT-PCR analysis revealed changes in HO-1 induction upon OR treatment. First, we measured the expression of HO-1 3, 6, 12, and 24 h after 1000 μg/mL OR treatment. HO-1 protein and mRNA expression were highest at 6 and 3 h, respectively (Figure 3C). As shown in Figure 3D, OR induced HO-1 protein and mRNA expression at concentrations of 500 and 1000 μg/mL in a concentration-dependent manner.

### OR inhibits NF-κB pathway activation in macrophages upon LPS stimulation

**Figure 4:**
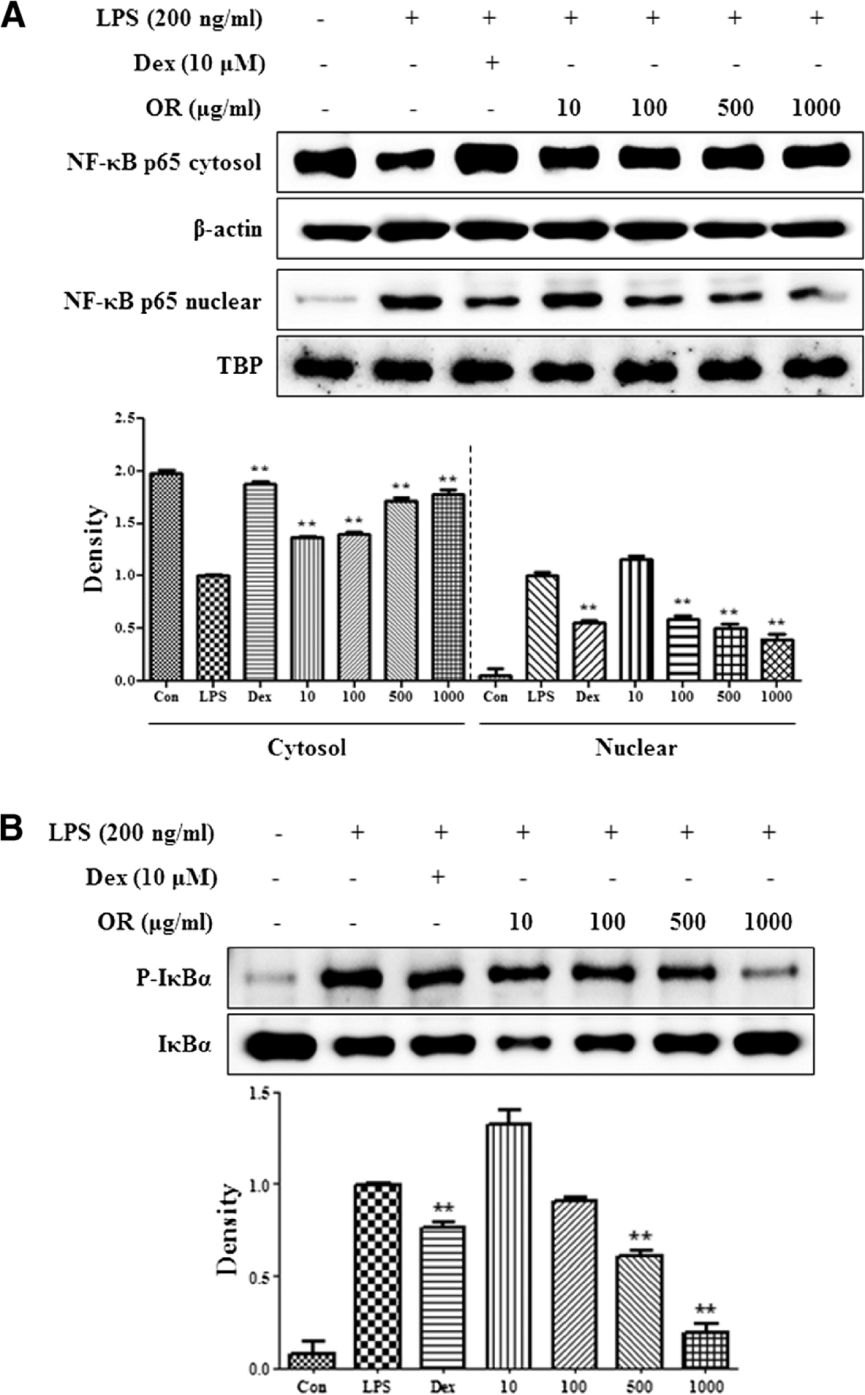
**Inhibitory effect of OR on (A) NF-κB translocation into the nucleus and (B) IκBα phosphorylation.** The cells were treated with LPS alone or with LPS and OR for 30 min (IκBα) or for 1 h (NF-κB). Protein expression in the cytosol or nucleus was determined by Western blot analysis. Data represent mean ± SE values of duplicate determinations from three independent experiments. *p < 0.01 and **p < 0.001 were calculated from comparisons the with LPS-stimulation value.

The NF-κB pathway is closely related to the production of inflammatory cytokines and iNOS. We examined the effects of OR on NF-κB activation by analyzing p65 translocation into the nucleus and the phosphorylation of IκBα in the cytosol. Western blot analysis revealed that 100–1000 μg/mL OR significantly repressed p65 translocation into the nucleus (Figure 4A). Also, as presented in Figure 4B, phosphorylation of IκBα was suppressed in a concentration-dependent manner. Less IκBα was consistently found in the presence of the same OR concentrations. These results suggest that OR effectively inhibits LPS-induced NF-κB pathway activation by blocking the nuclear translocation of NF-κB and IκBα phosphorylation.

### OR suppresses LPS-induced phosphorylation of MAPKs in RAW 264.7 cells

**Figure 5:**
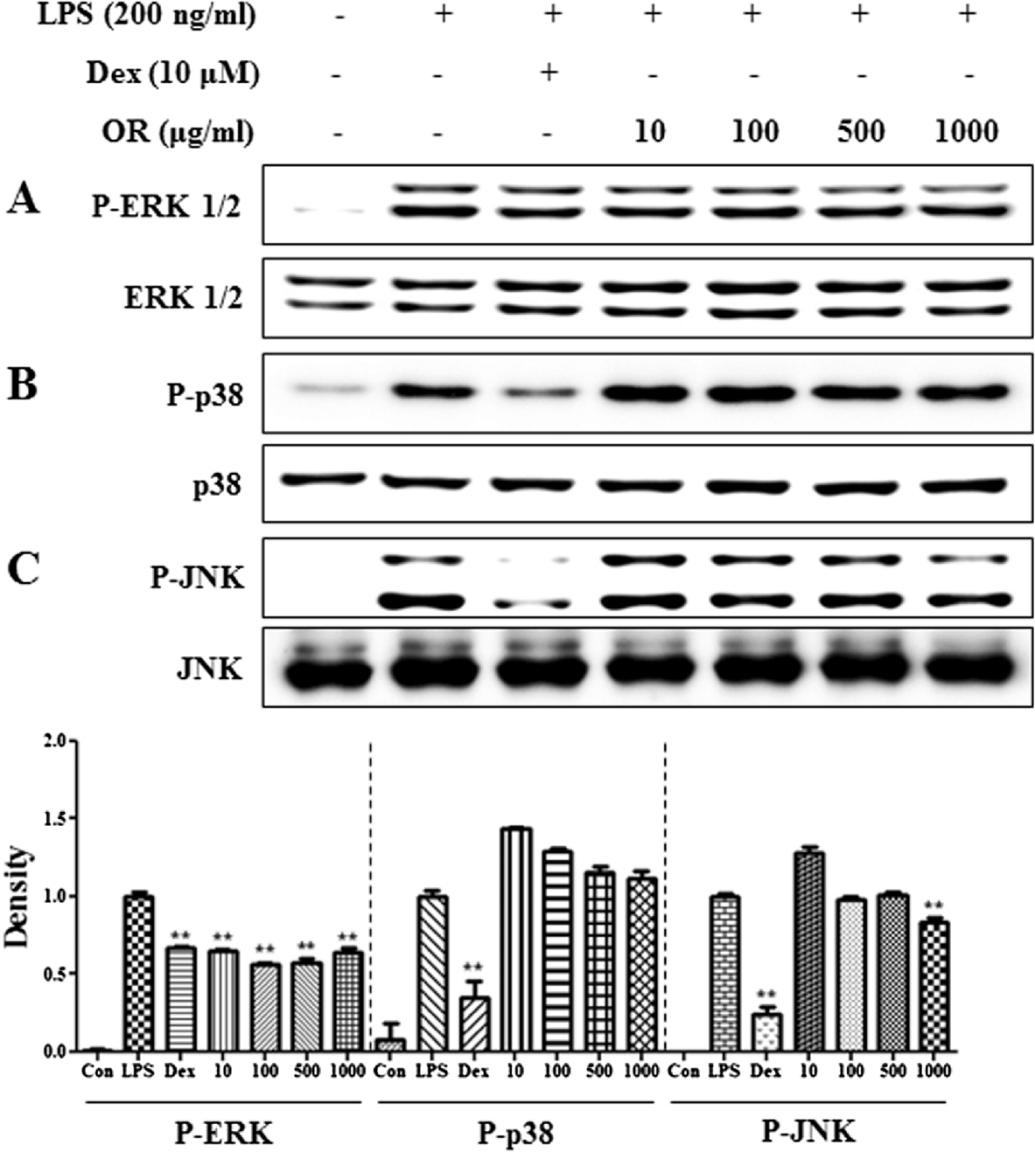
**Effect of OR on MAPK phosphorylation in macrophages: (A) ERK, (B) p38 and (C) JNK.** RAW 264.7 cells were treated with OR for 30 min before being incubated with LPS for 30 min. Cell lysates were analyzed by Western blotting using specific antibodies. Data represent mean ± SE values of duplicate determinations from three independent experiments. *p < 0.01 and **p < 0.001 were calculated from comparisons with the LPS-stimulation value.

Because MAPKs activated by phosphorylation play an important role in NF-κB pathway activation, we examined the inhibitory effect of OR treatment on activation of the MAPK pathway. We examined the phosphorylation levels of MAPKs, including ERK 1/2, p38, and JNK. When RAW 264.7 cells were stimulated with LPS in the presence of OR, the level of phosphorylated ERK MAPK was significantly decreased (Figure 5A). However, OR (1000 μg/mL) did not affect p38 or JNK activity (Figure 5B and C). And, we determined that the total forms of ERK, p38, and JNK were not affected by OR treatment.

### Selection of suitable wavelengths

**Figure 6:**
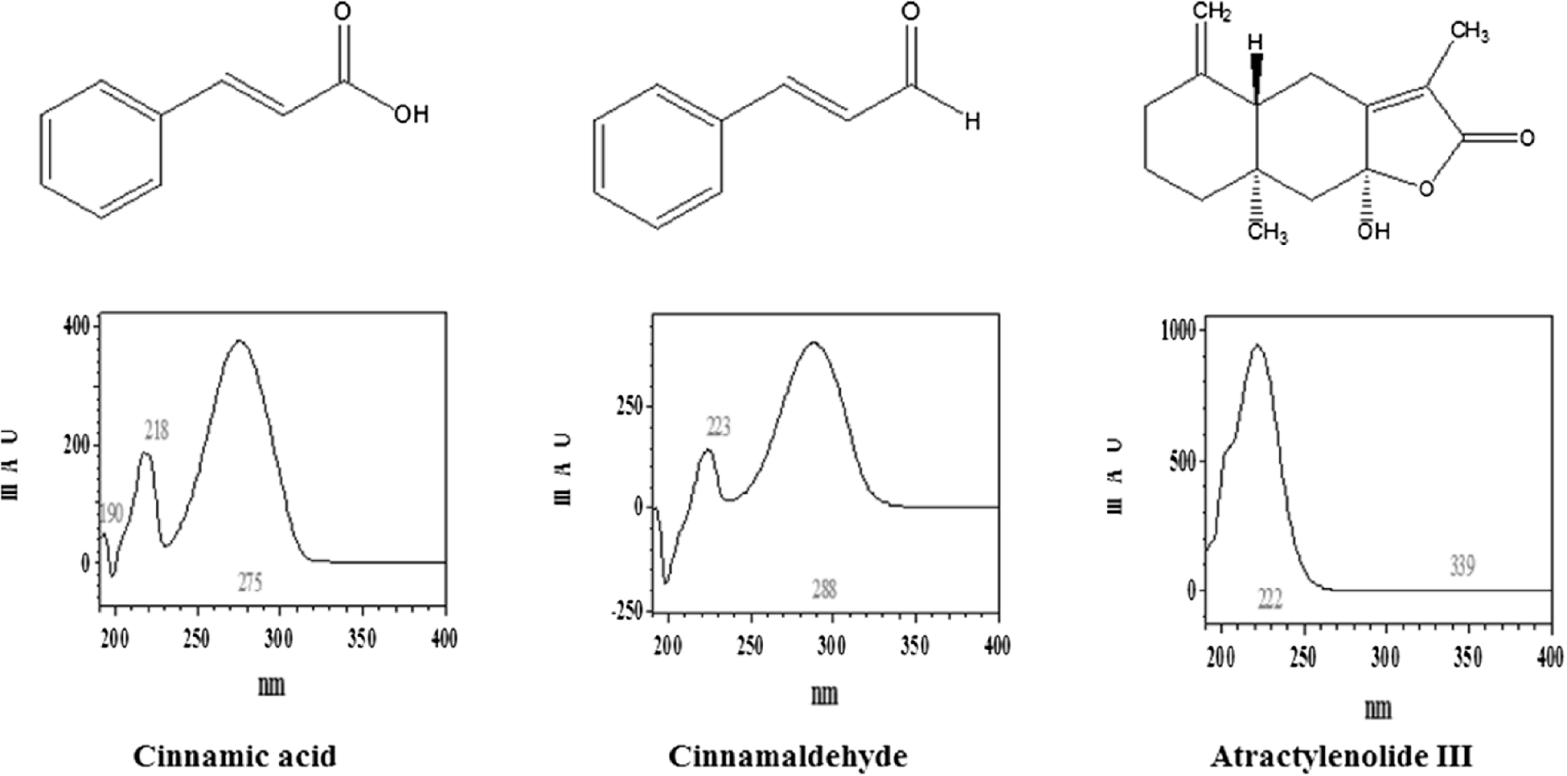
Chemical structures and HPLC DAD spectra of the main constituents of OR.

To find the optimum absorbance for each analyte, we obtained UV/VIS spectra at a range of 190–400 nm. The optimum absorbance for all analytes was determined to be 220 nm according to the individual absorbances: atractylenolid III, 227 nm; cinnamic acid, 223 nm; and cinnamaldehyde, 218 nm (Figure 6). The qualitative identification of three standards in OR was achieved using HPLC and was based on comparisons of UV wavelengths with those of the standard compounds.

### Analysis of the contents using HPLC

**Figure 7:**
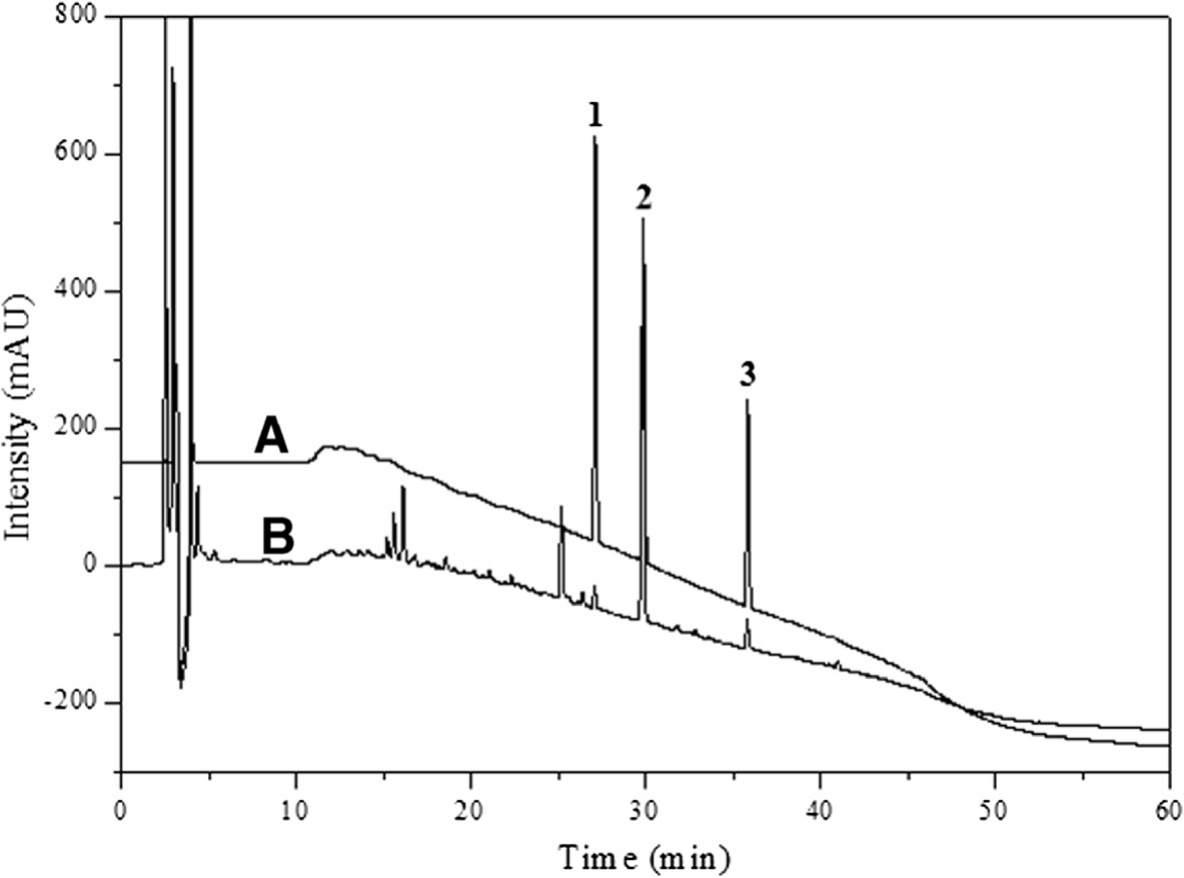
**HPLC chromatograms of the (A) standard mixture and (B) OR at 220 nm.** 1, Cinnamic acid, 26.96 min; 2, Cinnamaldehyde, 29.64 min; 3, Atractylenolide III, 35.55 min.

The peaks of the three standards appeared at 26.96 min for cinnamic acid, 29.64 min for cinnamaldehyde and 35.55 min for atractylenolide III. Figure 7 shows that the standard compounds and mixture sample were separated successfully and analyzed simultaneously. Identification of the certified OR compounds was based on comparisons of their retention times (t_R_) and chromatograms with those of the standard compounds (Figure 7).

## Discussion

Previous studies on natural herbs and herbal decoctions using *in vitro* and *in vivo* systems have been conducted to discover potential anti-inflammatory products. OR is an important formulation in oriental traditional medicine, and has been commonly used to treat symptoms associated with renal diseases in East Asia since ancient times. OR has protective effects against acute gastric mucosal injury and an inhibitory effect on the renin-angiotensin-aldosterone pathway [1,2]. Among the five herbs that making up OR, the anti-inflammatory effects of Atractylodes Rhizome White have been studied in RAW 264.7 cells [32]. The anti-inflammatory effects of cinnamon bark and *Alisma* rhizome have been studied in both *in vitro* and *in vivo* systems, and have been shown to have inhibitory effects on NF-κB activation [33,34].

In the present study, we demonstrated the anti-inflammatory activity of OR in RAW 264.7 murine macrophages stimulated with LPS. First, we determined that OR treatment did not result in cytotoxicity of RAW 264.7 macrophages; it did not affect cell viability up to a concentration of 1000 μg/mL. NO overproduction is associated with various inflammatory diseases [35,36], thus we investigated the inhibitory effects of OR on NO production induced by LPS stimulation. OR strongly suppressed NO secretion and inhibited iNOS expression and also suppressed COX-2 expression in a concentration-dependent manner. These results indicate that OR has inhibitory effects on the production of pro-inflammatory mediators.

The induction of HO-1 expression was due to a direct effect on iNOS expression [16]. Therefore, we investigated whether the inhibitory effect of OR on iNOS expression was associated with increased HO-1 production. We found that OR pretreatment at a concentration of 500 μg/mL or greater induced HO-1 expression in RAW 264.7 macrophages, and also determined that it affected the inhibiting efficacy of NO and iNOS production. This finding suggests that inhibitory effect of OR on NO production was influenced by not only blockade on activation of NF-κB and MAPKs pathways but also induction of HO-1 expression.

OR concentration-dependently suppressed the inflammatory cytokines TNF-α, IL-6 and IL-1β. NF-κB is a key transcriptional regulator associated with the cellular response to stimuli such as LPS [37-39]. Furthermore, it plays an important role in cell viability and the expression of various inflammatory factors including NO, inflammatory cytokines, and PGE_2_[40-42]. To investigate whether the inhibitory effect of OR on the expression of cytokines and inflammatory factors is associated with NF-κB pathway activity, we measured the effect of OR on NF-κB nuclear transcription. We found that OR concentration-dependently inhibited the nuclear transcription of p65 through the inhibition of IκBα degradation by LPS stimulation. These findings are consistent with previous studies showing that the NF-κB response drives the expression of iNOS, TNF-α, and IL-6 genes [43-45]. Because of many anti-inflammatory drugs repress the production of inflammatory mediators through inhibition of NF-κB activity, OR extract could be developed as anti-inflammatory agents.

Because MAPKs activated by LPS are related to iNOS expression in macrophages [46], we also examined the inhibitory effect of OR on the phosphorylation of MAPKs. OR significantly inhibited phosphorylation of ERK MAPK, but had a little effect on the phosphorylation of p38 and JNK MAPK. These results indicate that the inhibitory effect of OR on the phosphorylation of MAPKs is directly related to inhibition of NF-κB activation and reduction of inflammatory factor production in RAW 264.7 cells. In this study, we investigated whether OR have inhibitory activity on various inflammatory mechanisms including NF-κB, MAPKs and HO-1. As a results, OR shows strongly biological effect on various signaling pathways. This experiment design in vitro inflammation-related model was fundamental and comprehensive format in this field.

As shown in Figure 6, we identified three main components (cinnamic acid, cinnamaldehyde and atractylenolide III) in OR. A previous study reported that cinnamaldehyde has anti-inflammatory activities in vitro and in vivo [33]. Additionally, it was demonstrated that atractylenolide III inhibits LPS-induced TNF-α and NO production in macrophages [47]. These facts suggest that the anti-inflammatory activity of OR might be related to active components of OR, including cinnamaldehyde and atractylenolide III.

## Conclusions

In conclusion, OR had a strong inhibitory effect on NO secretion, inflammatory cytokines production and expression of iNOS and COX-2 in LPS-stimulated RAW 264.7 cells. These effects were due to inhibition of NF-κB activation through suppression of IκBα degradation and blockade of MAPK phosphorylation. Also, the influence of OR on HO-1 expression affected the suppression of inflammatory factors. These results suggest that OR could be developed as a new anti-inflammatory agent derived from natural products.

## Competing interests

The authors declare that they have no competing interests.

## Authors’ contributions

YCO, YHJ, JHH, WKC and JYM participated in the design of the study, YCO carried out the experiments, analyzed the data and wrote the paper. All authors read and approved the final manuscript.

## Pre-publication history

The pre-publication history for this paper can be accessed here:

http://www.biomedcentral.com/1472-6882/14/242/prepub
